# Incubation and Intuition in Creative Problem Solving

**DOI:** 10.3389/fpsyg.2016.01076

**Published:** 2016-07-22

**Authors:** Kenneth J. Gilhooly

**Affiliations:** ^1^Psychology Department, University of HertfordshireHatfield, UK; ^2^Department of Clinical Sciences, Brunel University LondonLondon, UK

**Keywords:** creativity, intuition, problem-solving, incubation effect, insight problem solving

## Abstract

Creative problem solving, in which novel solutions are required, has often been seen as involving a special role for unconscious processes (Unconscious Work) which can lead to sudden intuitive solutions (insights) when a problem is set aside during incubation periods. This notion of Unconscious Work during incubation periods is supported by a review of experimental studies and particularly by studies using the Immediate Incubation paradigm. Other explanations for incubation effects, in terms of Intermittent Work or Beneficial Forgetting are considered. Some recent studies of divergent thinking, using the Alternative Uses task, carried out in my laboratory regarding Immediate vs. Delayed Incubation and the effects of resource competition from interpolated activities are discussed. These studies supported a role for Unconscious Work as against Intermittent Conscious work or Beneficial Forgetting in incubation.

“Intuition: the power of the mind by which it immediately perceives the truth of things without reasoning or analysis; a truth so perceived, immediate, instinctive knowledge or belief.

Latin, *in*, into, upon, and *tueri*, *tuitus*, to look.” *The Chambers Dictionary, 9^th^ Edition*, 2003, p. 778. Edinburgh: Chambers Harrap.

Creative problem solving involves the production of approaches and solutions that are novel to the solver even if not historically novel (Boden, 2004). Explaining the generation of personally novel solutions is an unresolved issue for the psychology of thinking and problem solving. Sometimes, problems seem to be solved by an immediate intuition or insight (e.g., Salvi et al., 2016) but, with difficult problems, a period of conscious analysis is usually needed, even if it does not directly lead to solution and the problem is set aside before solution. Why might setting a problem aside facilitate solution? One popular explanation is that setting creative problems aside for a period can allow unconscious processes to generate solution ideas, which are then experienced, either as spontaneous breakthroughs into consciousness while attention is focussed on other matters, or as very rapid solutions on returning to previously intractable problems. These solutions occurring apparently rapidly and without awareness of intermediate steps, will be experienced as akin to the dictionary idea of an intuition as a truth (a solution in this case) perceived without reasoning or analysis.

The value of setting a problem aside for facilitating solutions has been a concern of theorists in the area for at least the past 100 years. Wallas (1926, p. 80) drew on Poincaré’s (1910) earlier analysis of mathematical creation and labeled the stage in which a problem is not consciously processed as “Incubation.” (It is noteworthy that Poincaré himself did not use the term “Incubation” in his 1910 paper, although he reported four examples of incubation periods from his own experience of creative work in mathematics). In Wallas’s analysis, Incubation is proposed as a useful stage after conscious Preparation but preceding Illumination (or Inspiration) and Verification. Clues to processes underlying creative thinking should be found from analyses of when and why Incubation can be useful. Subjective reports by acknowledged creative thinkers over many areas of work have supported the existence of incubation phenomena (e.g., Poincaré, 1910; Ghiselin, 1952; Csikszentmihalyi, 1996). However, since such personal reports have often been given many years after the events described, the reliability of such reports is highly questionable. For example, frequently cited accounts by Coleridge (composition of poem *Kubla Khan* in a dream), Mozart (complete compositions coming to mind without error) and Kekulé (discovery of benzene ring in a dream) have proven to be false (Weisberg, 2006, pp. 73–78). Poincaré (1910) himself based his own analysis of creative thinking on self reports of problem solving episodes he had experienced nearly 30 years previously. This is actually rather curious, as Poincaré was an active researcher in mathematics at the time of making his analysis of creative thinking and could presumably have drawn on more recent episodes which would be less susceptible to recall problems. However, after Poincaré (1910) and Wallas (1926), who had relied on their own introspections and on subjective reports by others (e.g., Wallas drew on daydream reports by Varendonck, 1921), a substantial body of experimental work research has been carried outusing both (a) *insight* problems, in which t the solver has to develop a re-structuring of the task to reach a unique solution and (b) *divergent* problems, that have no single unique solution but in which many novel potential solutions are to be generated. A typical divergent task, often used in research studies, is the Alternative Uses Task. In this task, participants are to produce as many uses as they can which are different from the normal use in response to one or more everyday items, such as a house building brick, a coat hanger, a pencil, a paperclip, and so on (Guilford, 1967; Guilford et al., 1978; Gilhooly et al., 2007).

Early work on incubation used a laboratory paradigm, known as the *Delayed Incubation Paradigm*, in which participants work on the target problem for an experimenter set preparation time before being given an *interpolated activity* different from the target task for a setincubation period before returning to the target problem for a set post-incubation work time. Performance in the incubation condition is compared with that of the control condition in which participants work without a break on the target task for a time equal to the sum of preparation and post-incubation conscious working times in the incubation condition. A recent alternative, the *Immediate Incubation paradigm*, has an interpolated task *immediately after* the instructions on the main problem *before* any conscious work has been undertaken on that problem, followed by uninterrupted work on the maint problem for a set time (Dijksterhuis and Meurs, 2006).

## Delayed and Immediate Incubation Effects

There is now considerable evidence from laboratory studies for the benefits of Delayed Incubation, i.e., that setting a problem aside after a period of work is beneficial (see Dodds et al., 2012, for a qualitative review). A quantitative meta-analysis by Sio and Ormerod (2009), of 117 studies identified a positive effect of Delayed Incubation, where the overall average effect size was in the low-medium band (mean *d* = 0.29) over a range of insight and divergent tasks. Sio and Ormerod’s review also revealed that the benefits of an incubation period are greater when participants are occupied by an undemanding interpolated task than when they engage in a demanding interpolated task or no task at all. Overall, from narrative reviews and meta-analysis, it can be concluded that the basic existence of Delayed Incubation effects is clearly established, especially for divergent problem solving.

Concerning the effectiveness of Immediate Incubation opportunities, Dijksterhuis and Nordgren (2006) found that better performances when Immediate Incubation occurred after decision problems or divergent tasks were initially presented. Indeed, Nordgren et al. (2011) reported that Delayed Incubation resulted in better decisions than Immediate Incubation and both types of incubation were beneficial relative to No Incubation.

A meta –analysis (Strick et al., 2011) of 92 decision studies found a significant beneficial aggregate effect size of *g* = 0.224 for Immediate Incubation. Their results also pointed to a number of moderating factors, for example, beneficial effects were greater, with more options, with shorter presentation times, with shorter incubation times and with induction of a configural mindset vs. a feature based mindset.

In creative divergent tasks Dijksterhuis and Meurs (2006), reported that responses were more creative on average, when the divergent task instructions were followed immediately by a short distracting task before producing uses for a brick, compared to a control condition. We may note that the instructions in this study did not ask for unusual uses, which is the norm in divergent thinking tasks, and so it is not clear whether participants had the goal of being creative. Participants may have been reporting infrequent uses, that they happened to know, rather than generating uses novel to them at the time of test. Raters tend to score infrequent responses as creative, although such uses may have been pre-known and therefore could reflect memory retrieval rather than generation of subjectively novel responses (Quellmalz, 1985). However, Gilhooly et al. (2012) using more standard instructions with a stress on unusual uses found a stronger beneficial effect of Immediate Incubation than of Delayed Incubation with both incubation effects being superior to control effects, scored for fluency and novelty of responses. Thus, the benefit of immediate incubation was also found when the task involved novelty (Gilhooly et al., 2012) as well as fluency (Dijksterhuis and Meurs, 2006).

Zhong et al. (2008), applied the Immediate Incubation paradigm to the Remote Associates Task (RAT), in which solvers have to generate an associate common to three words (e.g., *cottage, blue, mouse*? Answer : *cheese*), and found that, Immediate Incubation activated solution words more on unsolved trials. compared to solution word activation on unsolved trials where that had been no Immediate Incubation.

Overall, it may be concluded from both meta-analyses (Sio and Ormerod, 2009; Strick et al., 2011) and from recent studies (Gilhooly et al., 2012, 2013, 2015) that incubation periods, whether delayed or immediate, do have beneficial effects. The main theories regarding mechanisms underlying incubation effects will now be outlined.

## Theories of Incubation Effects

### Intermittent Conscious Work

This approach proposes that participants carry out intermittent conscious work during the incubation period despite instructions to be fully engaged on the interpolated task used to fill the incubation period (Seifert et al., 1995, p. 82; Weisberg, 2006, pp. 443–445). Any conscious work during the supposed incubation period would help reduce the time required when the target problem was re-addressed – but conscious work on the target task would be expected to impair performance on the interpolated task. This theory has the merit of parsimony and essentially explains incubation away as not involving any special processes, such as intuitive unconscious thinking.

### Beneficial Forgetting

This view (e.g., Woodworth, 1938; Simon, 1966; Smith and Blankenship, 1991; Smith, 1995; Segal, 2004; see also, Dijksterhuis and Meurs, 2006) argues that “mental sets,” weaken during the incubation period. Such “beneficial forgetting” facilitates fresh starts or “set shifting” when the problem is taken up again after the incubation period. As well as decay and interference, misleading approaches may conceivably be weakened through inhibition as proposed in the theory of retrieval-induced forgetting (Anderson et al., 1994; Storm and Angello, 2010). Segal (2004) proposed a variant (known as “Fresh Look”) in which simply switching attention away from the main task allowed a new start, with no forgetting or unconscious work proposed. The Fresh Look view does not predict effects of Immediate Incubation because with in that condition, there is insufficient opportunity for sets or fixations to develop that need to be forgotten to enable later progress.

### Unconscious Work

On this account incubation effects involve active, but unconscious, or intuitive processing. The term “unconscious work” seems to first appear in the problem solving literature in Poincaré’s (1910) paper (p. 328). Related phrases such as “non-conscious idea generation” (Snyder et al., 2004) and “unconscious thought” (Dijksterhuis and Nordgren, 2006; Ritter and Dijksterhuis, 2014) are also used in the literature, but I will use the phrase “unconscious work” throughout the present paper.

Theoretically, what form might unconscious work take? For example, could unconscious work be exactly like conscious work, but with just one difference, namely that it is carried out without any conscious awareness? Or is unconscious work better thought of as some form of automatic spreading activation along associative links, as against a conscious rule or strategy governed activity? Wallas (1926) proposed the idea of spreading “associative chains” as being active during incubation, which can be seen as anticipating modern ideas of spreading activation. Poincaré (1910) argued for quite specific mechanisms of automatic idea generation and selection tailored to his domain of interest which was mathematical creation. Both Poincaré and Wallas argued that the suddenness of Illumination or Inspiration coupled with the feeling of confidence in the sudden insight arose from prolonged unconscious work. Wallas’s analysis is often labeled as a Four Stage theory, incorporating Preparation, Incubation, Illumination, and Verification, but he also proposed a sub-stage of Illumination which he dubbed “Intimation” (Wallas, 1926, p. 97). This sub-stage is often overlooked in discussions of Wallas’s analysis, although Wallas considered it was important, practically and theoretically (see also, Sadler-Smith, 2015, for an extended discussion of Intimation in Wallas’s model). Intimation is the moment at the very start of the Illumination period when the solver becomes aware that a flash of success is imminent. Theoretically, Wallas saw Intimation as reflecting increasing activation of a successful association train which was about to become conscious. Thus, Intimation was consistent with the view that Incubation involved unconscious work. Practically, Wallas felt it was important that the solver recognize the Intimation feeling and desist from distracting activities to allow the solution to continue rising into consciousness. Overall, unconscious work has long been favored as a possible explanation of incubation effects. The question of what specific processes might be involved in unconscious work will be considered further in the Theoretical Discussion section.

The possible mechanisms indicated above are not mutually exclusive (or exhaustive). Delayed Incubation could involve all three suggested mechanisms, with some intermittent conscious work taking place when attention strays from the distracting task during the incubation period and with some beneficial forgetting and unconscious work also occurring when the solver is consciously processing to the distracting incubation task. However, a beneficial effect of Immediate Incubation would not be consistent with the Beneficial Forgetting hypothesis in that there is not time in the Immediate paradigm for sets or misleading directions to be established, but the Immediate paradigm would permit some intermittent conscious work and/or some unconscious work.

## Theories of Incubation: Empirical Evidence

### Intermittent Work

As a check for intermittent conscious work during an incubation period, performance on the interpolated task, during incubation, should be compared with performance by a control group using the interpolated task as a stand-alone activity. Impaired interpolated task performance during incubation would be consistent with the hypothesis of some conscious work on the target task during incubation. The argument here being that intermittent conscious work represents a diversion of resources away from the interpolated task and that should impair performance on the interpolated task. Although this may seem a basic methodological check for intermittent conscious work, it does not appear to have been carried out (Sio and Ormerod, 2009; Dodds et al., 2012) until quite recently. In particular, Gilhooly et al. (2012, 2015) incorporated suitable checks for intermittent conscious work on a target divergent thinking task during the incubation period. In an experiment involving delayed and immediate incubation and two different interpolated activities (Gilhooly et al., 2012), there was no evidence of impairment to the interpolated incubation period tasks (which were mental rotations and anagram solving) as a result of the tasks being carried out during incubation periods, as against being carried out as stand-alone tasks in control conditions. These studies also found positive incubation effects, despite a lack of evidence for intermittent conscious work. If anything, the trends in the data were the opposite of those that would be predicted by the intermittent work hypothesis. Mental rotation and anagrams were somewhat (but not significantly) facilitated by being carried out as distractor tasks during incubation. None of the one tail predictions of the intermittent conscious work hypothesis were upheld. An additional analysis examined the correlations between performance scores on the interpolated tasks and post-incubation scores on the target, divergent thinking task. The Intermittent Work Hypothesis would predict negative correlations in that the more attention given to the interpolated task, the better the interpolated task scores would be, and the worse would be the target task scores. Over eight Pearson correlations examined, two were negative and six positive; the average Pearson correlation between target task and interpolated task performance measures was 0.11. Only one correlation was significant (*r* = 0.36, *p* < 0.05, two tail) and this was in the direction opposite to that predicted by the Intermittent Work Hypothesis. This analysis of correlations between interpolated task and target task performance measures thus did not support the Intermittent Work hypothesis. A later study (Gilhooly et al., 2015) using a target divergent thinking task and mental rotations as the interpolated task in a delayed incubation paradigm, also found no impairment in the interpolated task relative to controls. Indeed, mental rotations were significantly better performed as an interpolated task as against as a stand-alone task, contrary to the Intermittent Work Hypothesis.

In a related study, Baird et al. (2012), using thought monitoring techniques, found that frequency of target task related intermittent thoughts during incubation was not related to quality of performance after the incubation period. So, it seems that even if intermittent thoughts about the target task occurred they were ineffective and did not explain the beneficial effects of incubation. In conclusion, from Baird et al. (2012) and Gilhooly et al. (2012, 2015), it seems safe to rule out the Intermittent Work explanation of incubation effects.

### Beneficial Forgetting

On this view, solvers often develop initial approaches that are misleading and become fixated on these approaches. A break allows such tendencies to become weaker and so a fresh start is possible when the problem is resumed after an incubation break.

Smith (1995) investigated this possibility using word problems presented either with helpful or with misleading cues. After failures to solve, participants were given breaks of varying lengths and then on returning to the task tried to recall the cues and to solve. In the case of misleading cues, participants were more likely to solve when they had forgotten the cues and likelihood of forgetting increased with length of the break. The results thus supported the idea that beneficial forgetting of misleading information could be a factor underlying incubation effects.

Segal (2004) examined a variant of the Beneficial Forgetting approach which may be labeled the Fresh Look hypothesis. On this variant, simply switching attention from the target task is enough and length of break is not important. His study involved a spatial insight problem, in which a square has a parallelogram superimposed on it and the task is to find the sum of the areas of the two shapes. The problem is made easier when the solver realizes that the shapes can be restructured as two equal sided right angle triangles which, if slid, form a rectangle whose area is easily calculated. Participants engaged in this target task until they felt they were experiencing an *impasse.*

After *impasse*, participants were given 4 or 12 min on either a demanding verbal task (crossword) or undemanding task (browsing through newspapers) and then returned to the main task for up to 6 more min.

Results indicated significant benefits for incubation break v. no break, but no effects for length of break or for the demandingness of the activity during the break. Segal argued that these results were consistent with a the Fresh Look view, that simply removing attention from the target task was sufficient and that it was not important what was done in the incubation period or how long it was. This study thus supports a role for attentional shifting as a mechanism for Delayed Incubation. Together, Smith (1995) and Segal (2004) are consistent with a role for Beneficial Forgetting in the Delayed Incubation paradigm.

### Unconscious Work

In contrast to Smith (1995), Segal (2004), and Dijksterhuis and Meurs (2006) argued that in the Immediate Incubation paradigm, the Beneficial Forgetting approach may be ruled out as there is no period of initial work in which misleading fixations and sets could be developed. Thus, if Immediate Incubation is shown to be effective, the unconscious work hypothesis must remain in contention for Immediate Incubation effects at least and would also be a candidate explanation as one possible mechanism for Delayed Incubation. Dijksterhuis and Meurs (2006) took the beneficial effects of the Immediate Incubation paradigm on a divergent task in their Experiment 3 as support for the role of unconscious work in incubation. However, as already mentioned, the task in this study did not clearly meet the usual criteria for a creative task and the scoring did not distinguish infrequent from genuinely novel responses. Hence, this study did not unequivocally address creative thinking as against free recall of possibly rare but previously experienced events from episodic and semantic memory.

Gilhooly et al. (2012) using explicit instructions to generate novel responses did find that both delayed and immediate incubation were effective in the Alternative Uses task and that immediate incubation produced more facilitation than delayed incubation. These results were consistent with a role for unconscious work in divergent thinking, particularly for Immediate Incubation, to which the Beneficial Forgetting approach is not applicable.

Snyder et al. (2004) investigated the role of unconscious work in the Delayed Incubation paradigm using a surprise return to the target task. In this case, beneficial effects of incubation emerged, consistent with the view that an automatic continuation of work but unconsciously may have occurred after the task was set aside. We may note that Snyder et al.’s (2004) task required simply production of uses for a piece of paper as against novel uses. Thus, this study did not necessarily require creative thinking as against recall of previously known uses.

The interpolated tasks used by Segal (2004) and by Dijksterhuis and Meurs (2006) were different in modality from the main tasks. Segal’s main task was spatial while the interpolated tasks were verbal and Dijksterhuis and Meurs’s study showed the opposite pattern in that their target task was verbal but the interpolated task was spatial. The similarity–dissimilarity relationship between target and interpolated tasks could be important theoretically as the main competing hypotheses suggest different effects of similarity between target and interpolated tasks. If unconscious work is the main process then interpolated tasks similar to the target task should interfere with any unconscious work using the same mental resources and so lead to weaker (or even reversed) incubation effects when compared with effects of dissimilar interpolated tasks. The unconscious work hypothesis suggests that when it comes to incubation it would be helpful to “do something different” from the target task. On the other hand, a forgetting account would suggest that interpolated tasks similar to the target task would cause greater interference, which would lead to more forgetting of misleading approaches and thus enhanced incubation benefits.

Helie et al. (2008) explored the effects of different interpolated tasks on the *reminiscence* paradigm in free recall. This is relevant to our present concerns because the reminiscence paradigm is analogous to incubation, in that an initial free recall is followed by interpolated tasks for a set period and then the same free recall is attempted a second time. The reminiscence score is the number of items recalled on re-test that were not recalled on the initial free recall. Helie et al. (2008) found that the more executively demanding the interpolated tasks were, the lower were the reminiscence scores for picture recall These results fitted well with Helie and Sun’s (2010) Explicit–Implicit Interaction model which envisages unconscious implicit processes running in parallel with conscious explicit processes. Helie et al.’s (2008) result is consistent with the Unconscious Work hypothesis for incubation in that more demanding interpolated tasks will leave less resources available for unconscious work. However, Helie et al.’s (2008) focus was free recall from episodic memory rather than creative thinking, which requires novel combinations and so, although suggestive, and consistent with Unconscious Work, this result does not directly address creative thinking which is the focus of the present paper.

Ellwood et al. (2009) found a beneficial effect on number of responses post-incubation of a dissimilar interpolated task in a Delayed Incubation experiment. However, this study used a fluency of uses task rather than a novel uses task. Also, as Ellwood et al. (2009) pointed out, although their findings are consistent with an explanation in terms of unconscious work, an explanation in terms of selective relief of fatigue could also be invoked to account for the effects of similarity between incubation and target tasks. On this view, for example, a spatial Delayed Incubation task very different from a main verbal task could facilitate more recovery from fatigue specific to verbal processing than might an interpolated verbal task. Gilhooly et al. (2013) included tests of the effects of the similarity between incubation and target tasks in an Immediate Incubation paradigm, so that where fatigue as an explanation could be examined. The Gilhooly et al. (2013) study factorially varied incubation activities (verbal – anagram solving vs. spatial – mental rotations), used either a clearly creative verbal divergent task (alternate uses) or a clearly spatial divergent task (mental synthesis) and both divergent tasks were scored for novelty as well as fluency. Significant incubation effects were found, but of most interest were the interactions, in that spatial incubation benefitted verbal divergent thinking more than did verbal incubation activity and verbal incubation activity benefitted spatial divergent thinking more than did spatial incubation activity. These results supported a role for unconscious work during incubation periods in creative thinking tasks and did not support the hypotheses that incubation effects are due to Beneficial Forgetting or attention shifting. The Beneficial Forgetting account predicted the opposite pattern of facilitation (i.e., that similar incubation and target tasks would be more beneficial than different modality incubation and target tasks).

## Theoretical Discussion

From recent research discussed above relating to the three main explanations for incubation effects, viz., Unconscious Work, Intermittent Work, and Beneficial Forgetting, it seems that given the effectiveness of Immediate Incubation, in which sets are unlikely to have been developed, the Beneficial Forgetting hypothesis can be ruled out for immediate incubation at least. In addition, Gilhooly et al. (2012, 2015) found no support for the idea of Intermittent Work, from studies in which suitable control conditions were included. Unconscious Work thus remains as the best candidate explanation for the effects of Immediate Incubation periods and it handles the effects of similarity between incubation and target task Gilhooly et al. (2013). Gilhooly et al. (2013) found that Delayed Incubation was beneficial, but less so than Immediate Incubation in a divergent thinking task (Alternative Uses). It could be that in Delayed Incubation, sets do build up during the initial period of conscious work, and are then reduced by Beneficial Forgetting, after which useful unconscious work could come into play. In contrast, with Immediate Incubation, there are no sets to be overcome and beneficial unconscious work can start sooner than in the Delayed paradigm leading to better performance than with Delayed incubation. Overall, however, the Unconscious Work hypothesis is in contention for both Delayed and Immediate Incubation.

However, the question still arises of what processes might be involved in unconscious work? Could unconscious work processes be identical toe conscious work processes with the sole difference that they are executed without conscious awareness? This issue will now be addressed.

### Unconscious Work?

Conscious work is generally rule or strategy governed. Could unconscious work also be rule governed? Poincaré (1910, p. 329) considered the possibility of a “subliminal self” that worked in the same way as the conscious self, but without consciousness, and might even be a superior “self” since it could find solutions that evaded the conscious mind. Kounios and Beeman (2015) illustrate this notion of a subliminal self by supposing that a man has the job of solving long anagrams during office hours. Suppose the person concerned works systematically all day, on the day shift, from 9 am to 5 pm, trying to solve say, “iaiaeiaeiiamsnrtnmhslbtssdtn,” but when he leaves at 5 pm it is still not solved. Another worker takes over and continues the systematic search on the night shift, from where the first worker left off. At 7 pm the night shift worker phones through to the day shift worker with the answer (cf., insight) saying “It’s “antidisestablishmentarianism!””. In this example, the second shift worker represents the unconscious and works just the same way, using systematic search, as the day shift worker; but, the day shift worker is not aware of the night shift worker’s activities until the answer is phoned through.

To explore further the idea that unconscious work might be a subliminal version of conscious work let us consider conscious processing in the Alternate Uses task. This was addressed in a think aloud study of the Brick Uses task by Gilhooly et al. (2007) in which it was found that participants used strategies, such as scanning the target object’s properties (“Bricks are heavy”) and using the retrieved properties to cue and infer uses from semantic memory (“Heavy objects can hold down things like sheets, rugs, tarpaulin and so on, so a heavy brick could do those things too”). Could unconscious work essentially duplicate this form of conscious work but with no awareness. As we have argued previously (Gilhooly et al., 2012, p. 976).

“The standard view in cognitive science is (a) that mental contents vary in activation levels, (b) that above some high activation level mental contents become available to consciousness, (c) that we are conscious of only a limited number of highly activated mental elements at any one time (that is, the contents of working memory) and (d) that strategy or rule based processing, as found in Gilhooly et al.’s (2012) think aloud study, requires such highly activated (conscious) material as inputs and generates highly activated (conscious) outputs.”

On the standard view then, conscious work requires the highly activated contents of working memory and highly activated material is necessarily in consciousness. Overall, it seems impossible that unconscious processes could really be exactly like conscious processes in every respect except that of being conscious. For example, using the rules of arithmetic and temporary working memory storage processes to multiply two 3 digit numbers (e.g., 364 × 279 = ?) is surely impossible without highly activated representations in working memory of the numbers, goals, and intermediate results. The short term representations involved in mental arithmetic would seem to be necessarily conscious. It seems impossible to carry out unconscious multiplication of two or three digit numbers. (With practice of course, one can learn and store three digit multiplication results in long term memory which can be directly retrieved by a type of unconscious process. However, this t is not mental multiplication). Poincaré (1910, p. 334) made a very similar point when he wrote “It never happens that the unconscious work gives us the result of a somewhat long calculation *all made*, where we only have to apply fixed rules.” In conclusion, the idea that unconscious work or thought processes could be just the same as conscious work processes with the sole difference that they lack awareness of any mental content, seems unlikely.

However, a challenge to this conclusion has been recently put forward by Hassin (2013) who argues in favor of what he labels a “Yes, It Can” (YIC) principle. According to YIC, unconscious processes can perform the same fundamental, high level functions that conscious processes perform. While it would be generally accepted that the elementary (fundamental?) component processes in carrying out 364 × 279 = ?, are unconscious (e.g., the first step of 364 × 279 is likely to be 9 × 4 = 32, which involves a direct retrieval process that occurs without conscious concomitants in adults practiced in basic multiplication at least) and many such steps and processes are needed, yet precise results need to be held in working memory and precise goals need to be formulated in an organized way (executive processes) all of which seems impossible without mental contents activated to conscious levels. Hassin cites some experiments (Sklar et al., 2012) which appear to show priming in subliminally presented additions and subtractions involving two and even three digits. However, these are far from the long calculations with intermediate results that Poincare discussed as difficult for the subliminal self. Exact calculations cannot realistically be made purely by priming which would activates associatively related numbers and not just the correct ones which are needed at every step of a long calculation if it is to be successful. Similar points apply to all types of problem solving which require multiple steps to be carried out and multiple intermediate results to be held along the way between presentation and solution.

Assuming unconscious work cannot actually be just the same in terms of processing steps as conscious work, of what then, might unconscious work consist?

Poincaré (1910, p. 333) drew on Epicurus’s (341–270 BC) ancient-world theory of atoms as having hooks so that these elementary building blocks of nature could combine with each other. He imagined ideas like hooked atoms hanging on a wall before relevant ideas/atoms are set in motion during Preparation and continue in motion during Incubation. As with molecules of a gas in a container, the atoms/ideas collide at random and sometimes the hooks snag and a new combination is formed. The atoms initially set in motion can strike atoms at rest and may combine with them. This would represent initial ideas being combined with new ideas so that the products of random combination would always have some relation to the starting conditions of the problem.

Campbell (1960) drew on a range of pre-cursors of his view who had stressed the role of extensive trial-and-error in creative work (Bain, 1874; James, 1880) and he was strongly influenced by Poincaré (1910). Campbell argued that creative problem solving involves a quasi-random generation of associations between mental elements (“Blind Variation”) to produce novel combinations of ideas, some of which may be useful and so be subject to Selective Retention. This approach draws an analogy with biological evolution in which random changes in genetic material lead to changes in organisms, some of which are useful and hence retained by natural selection. Similarly, it is argued that ideas are modified in creative problem solving in ways which are blind to the final solution and only by chance lead ultimately to modifications that solve the problem and are retained for future use. Campbell (1960) quoted extensively from Poincaré’s (1910) account of creative thinking in mathematics, as involving extensive quasi-random search, although Campbell did not stress any special role for unconscious processing. His concern was very much with the role of blind trial-and-error, whether carried out at a conscious or an unconscious level. It could be argued that Campbell saw productive conscious creative thinking as like the unconscious work proposed by Poincaré (1910).

Simonton (1995, 2003) developed Campbell’s ideas and used the notion of “mental elements” which are similar to Poincaré’s (1910) “hooked atoms.” However, unlike Campbell, Simonton stresses the role of unconscious processes which lead to new combinations, some of which are retained and selected to enter consciousness on the basis of their “stability.”

In terms of current approaches to cognitive processing, how might novel combinations come about? Parallel spreading activation processes in a semantic network could lead to remote and unusual associations (Jung-Beeman et al., 2004). One specific proposal is that of Helie and Sun’s (2010) Explicit–Implicit Interaction model. In this model, incubation is regarded as involving unconscious, implicit, stochastic associative processes that demand little attentional capacity in contrast with conscious explicit rule governed attentionally demanding processes that run in parallel. In this model, activation spreading through implicit networks during incubation periods leads to novel associations which could facilitate later work when conscious processing resumes and the explicit level processes and knowledge interact with the implicit level processes and knowledge. The model does not seem to deal with incubation leading to a breakthrough of solutions into consciousness without an explicit return to the task. According to Dijksterhuis and Nordgren’s (2006) Unconscious Thought Theory (UTT), unconscious thought, or work, is parallel, bottom-up, inexact, and divergent; whereas conscious thought is, serial, exact, and convergent. Thus, the characteristics of unconscious thought, as envisaged by UTT are consistent with incubation effects.

Overall, there is general agreement among many theorists that unconscious thinking, or unconscious work, in the form of implicit associative processes involving spreading activation [similar to Wallas’s (1926) concept of “associative trains”], is a possible explanation of incubation effects.

How might the suddenness of inspiration be explained? Both Poincaré and Wallas saw this feature of creative thinking as indicative of prolonged unconscious work that found a solution and delivered it to consciousness. However, here Poincaré identified a problem for the unconscious work account. How did the good idea become selected for promotion to consciousness? Poincaré was focussed on mathematical creation and he proposed that in this domain selection was based on the mathematician’s special intuitive sensibility to beauty in mathematics and further that the subliminal self possessed this intuitive sensibility. Poincaré’s theory, as stated in the 1910 paper, is narrow in solely addressing mathematical creation; generalization to other fields, such as poetry, music, physics, and so on, would require specific intuitive sensibilities to be proposed for those fields. An alternative possibility that has general applicability, is that when a problem is set aside, a goal representation remains active for extended time periods, although below the threshold for consciousness. The active goal representation would tend to boost activation flow into associated solution-relevant paths and when a solution combination of associations or a single relevant association became active, the solution and the goal representations would mutually activate each other in a positive feedback loop leading both to become conscious as their activations pass threshold levels. It is suggested that this rising activation (or “rising train of association” as Wallas put it) is experienced as Intimation. The present account has the benefit of automaticity and is parsimonious in not requiring special sensibilities to be invoked. The sub-threshold but active goal representation automatically does the work of selecting promising solution –relevant associations.

## Concluding Comments and Limitations

Overall, it can be concluded that the field, although still acknowledging the pioneering work of Poincaré and Wallas, has made considerable progress. The existence of incubation as a beneficial stage in creative thinking has been established through a large number of empirical studies (Sio and Ormerod, 2009), so that the field does not depend on potentially unreliable introspective accounts. New paradigms, such as Immediate Incubation have been established and have helped justify a role for implicit Unconscious Work. Theoretical ideas have been sharpened and refined and the joint effects of spreading activation and subconscious goal activation provide a candidate 9 explanation for insight or intuitive solutions following incubation. The approach put forward here, in terms of spreading activation and goal representations, is most applicable to relatively small scale but knowledge rich problems such as divergent thinking tasks. Further work is needed to develop the present approach for knowledge lean problems, such as laboratory insight problems on the one hand and for larger scale real life problems on the other hand.

## Author Contributions

The author confirms being the sole contributor of this work and approved it for publication.

## Conflict of Interest Statement

The author declares that the research was conducted in the absence of any commercial or financial relationships that could be construed as a potential conflict of interest.
